# Echocardiography and Cardiac Magnetic Resonance in the Assessment of Left-Ventricle Remodeling: Differences Implying Clinical Decision

**DOI:** 10.3390/jcm13061620

**Published:** 2024-03-12

**Authors:** Maciej Haberka, Monika Starzak, Grzegorz Smolka, Wojciech Wojakowski, Zbigniew Gąsior

**Affiliations:** 1 Department of Cardiology, School of Health Sciences (SHS), Medical University of Silesia, 40-635 Katowice, Poland; 2 Department of Internal Medicine, Angiology and Physical Medicine, Specialistic Hospital No. 2, 41-902 Bytom, Poland; 3 Department of Cardiology and Structural Heart Diseases, Medical University of Silesia, 40-635 Katowice, Poland

**Keywords:** cardiac magnetic resonance, echocardiography, left-ventricle function, left-ventricle mass, left-ventricle remodeling

## Abstract

**Introduction**: Transthoracic echocardiography (TTE) and cardiovascular magnetic resonance (CMR) are the most important modalities used in clinical practice to assess cardiac chambers. However, different imaging techniques may affect their results and conclusions. The aim of our study was to compare left-ventricle (LV) remodeling assessed using TTE and CMR in the context of various cardiovascular diseases. **Methods**: A total of 202 consecutive patients sent for an elective cardiovascular diagnosis were scheduled for a 2D TTE and CMR, performed within 2 weeks. The study group was divided and analyzed based on the clinical indications for CMR, including coronary artery disease, heart failure, native aortic valve regurgitation or paravalvular leak after aortic valve replacement, or cardiomyopathies. **Results**: The mean LV mass index (LVMi) values calculated using TTE were significantly larger (127.1 ± 44.5 g/m²) compared to the LVMi assessed using CMR (77.1 ± 26.2 g/m²; *p* < 0.001). The LV end-diastolic volumes assessed using TTE were underestimated for all the study patients (78.6 ± 43 mL vs. 100.5 ± 39 mL; *p* < 0.0001) and subgroups, but a statistical trend was observed in patients with cardiomyopathy. Those differences in single parameters led to differences in LV remodeling and the final treatment decision. CMR and TTE provided similar conclusions on LV systolic dysfunction in 68% of the patients. **Conclusions**: Our results showed that the greater the degree of LV remodeling and dysfunction, the greater the difference between the modalities. Therefore, CMR should be introduced into routine clinical practice, especially for patients undergoing LV remodeling, which may change clinical decisions in a considerable number of cases.

## 1. Introduction

Transthoracic echocardiography (TTE) and cardiovascular magnetic resonance (CMR) are the most important imaging modalities used to assess cardiac chambers and cardiac remodeling [1,2]. Two-dimensional (2D) TTE is the primary tool used to assess the left-ventricle (LV) ejection fraction (EF), LV volumes, mass, and hypertrophy in routine clinical practice. There are four types of LV remodeling, namely normal ventricle geometry, concentric remodeling, concentric hypertrophy, and eccentric hypertrophy [3]. However, echocardiographic formulas are based on linear measurements and rely on geometric assumptions of the LV shape [4]. Ultrasound contrast may be used in selected cases to improve endocardial border delineation in patients with suboptimal images taken using TTE. Contrast-enhanced apical views may provide greater LV volumes, which are closer to CMR results [3]. However, given the various chest-related causes of imperfect TTE images and the geometric assumptions used in echocardiography, contrast-enhanced TTE cannot replace CMR in the assessment of the LV cavity.

CMR is a modern and comprehensive imaging tool with high accuracy in the assessment of cardiac ventricle volume, function, and mass [5,6]. It provides a real measurement of the LV cavity instead of making geometric assumptions [2]. Contrast-enhanced CMR may provide multi-parametric results, including myocardial tissue characterization and myocardial injury [7]. Therefore, the role and position of CMR are stronger in terms of the consecutive cardiovascular guidelines of the European Society of Cardiology [8,9,10]. The precise assessment of the LV cavity and its remodeling have a fundamental role in image-guided individual therapy and the assessment of clinical prognoses [8]. Reference conclusions on LV enlargement, hypertrophy, and systolic dysfunction (EF) are crucial for patients with heart failure or valvular heart disease [9,10]. Data on the clinical utility of both modalities in assessing LV remodeling are limited. Therefore, our aim was to compare the assessment of LV remodeling using TTE and CMR in the context of various cardiovascular diseases.

## 2. Materials and Methods

**Figure 1:**
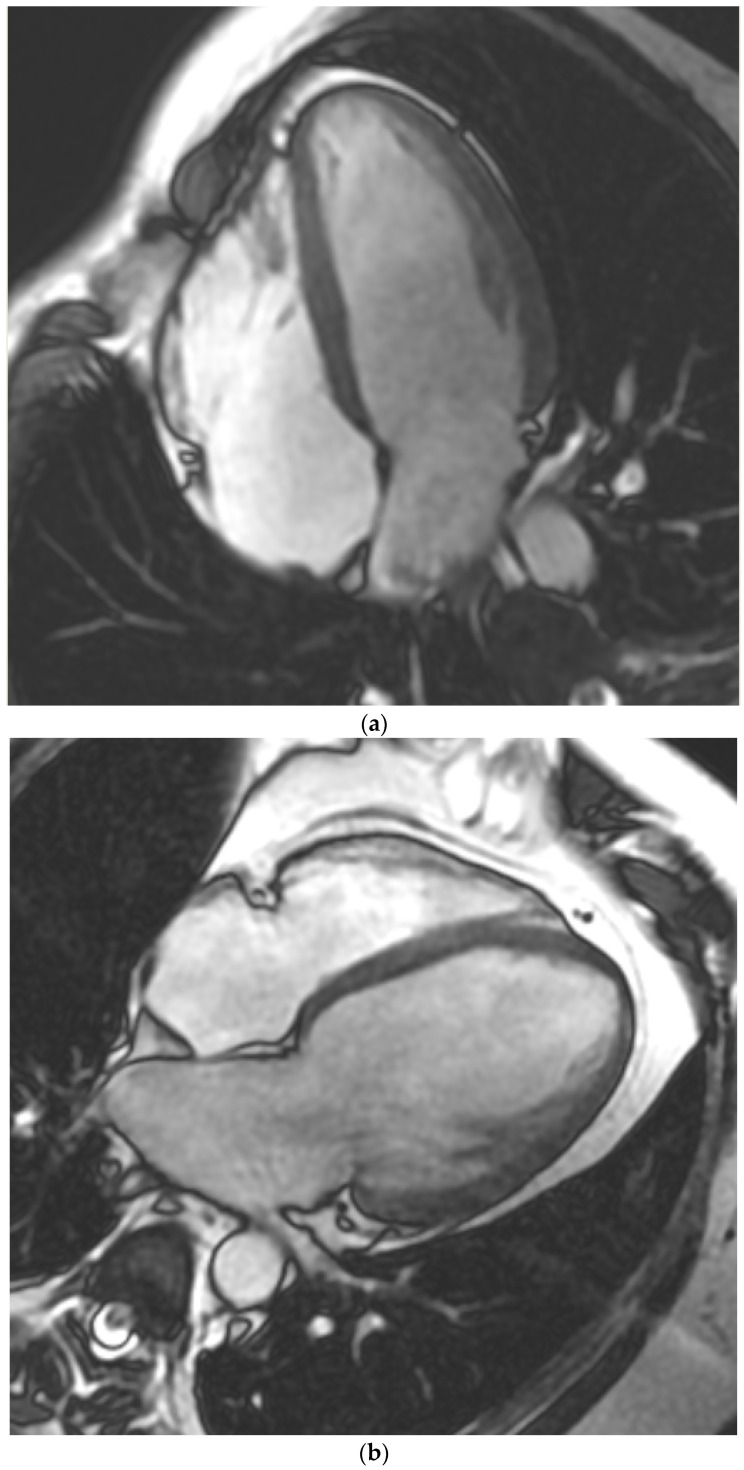

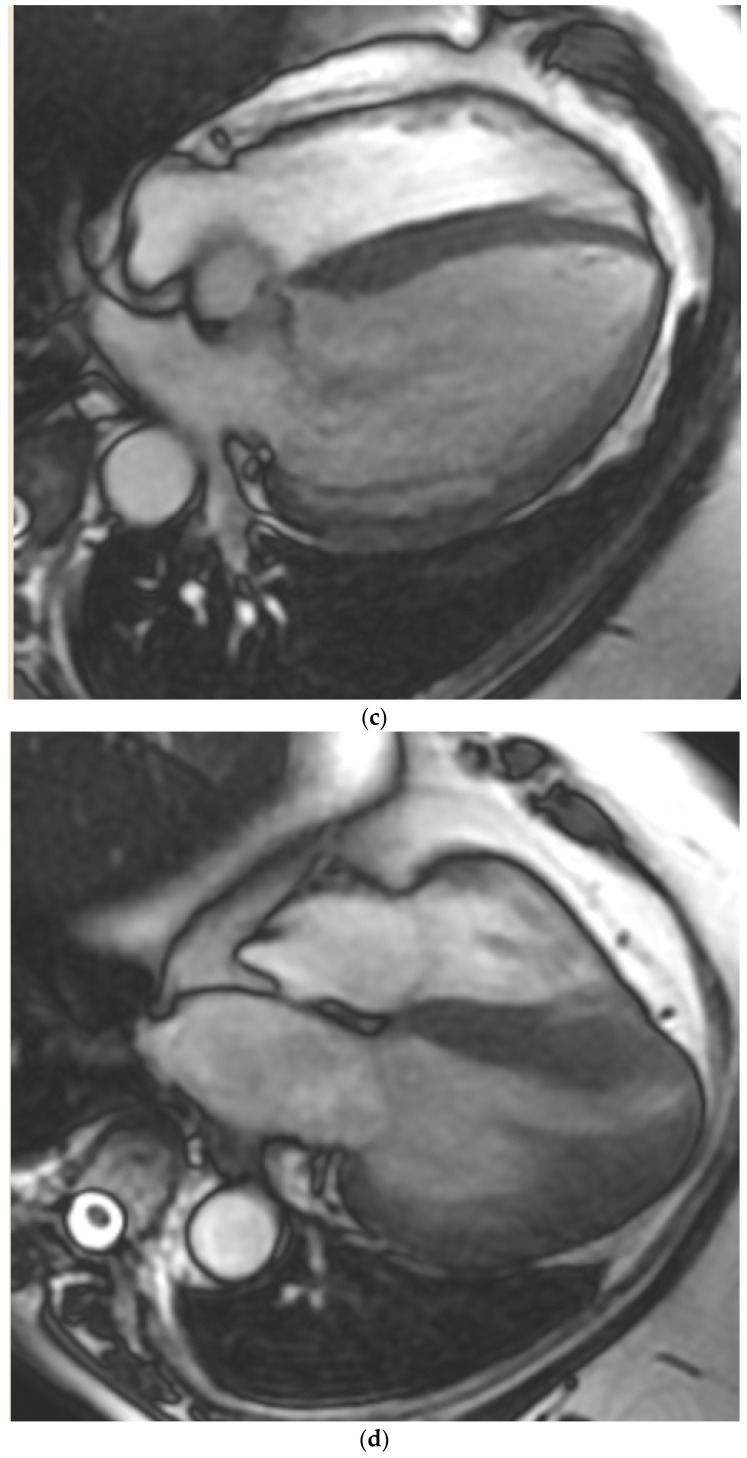
Cardiac magnetic resonance of left ventricles representative for Group 1 ((**a**)—normal cavity), Group 2 ((**b**)—heart failure), Group 3 ((**c**)—aortic regurgitation with dilated left ventricle), and Group 4 ((**d**)—hypertrophic cardiomyopathy).

We prospectively enrolled all patients scheduled for cardiovascular diagnostics (2017–2019) with both TTE and CMR performed within 2 weeks. All patients with the following unstable clinical conditions at the time of imaging or 8 weeks prior were excluded: acute coronary syndrome, acute pulmonary embolism, pulmonary edema, acute or decompensated heart failure, significant infections, or significant inflammatory diseases. The study group was divided and analyzed based on the following clinical indications for CMR: (1) coronary artery disease (CAD) in patients with preserved LVEF and without significant heart-valve disease, (2) heart failure (HF), (3) primary native aortic valve regurgitation (AR) or paravalvular leak after aortic valve replacement (PVL-AVR), or (4) cardiomyopathies (CM) (Figure 1a–d). The patients’ clinical characteristics were obtained from the hospital’s electronic medical records. All patients provided written informed consent to participate in the study. This prospective single-center study was performed in the Upper-Silesian Medical Center of the Medical University of Silesia in Katowice. Some of the study patients (within Groups 1 and 3) that were scheduled for CMR were included in our previous papers evaluating distinct aspects of cardiac imaging, which are not related to this study (quantification of paracardial fat depots or quantification of native and paravalvular aortic regurgitations) [11,12,13]. This paper presents results that have not been used or published before. The study was conducted in accordance with the principles of the Declaration of Helsinki and the local ethics committee. The study was approved by the ethics committee of the Medical University of Silesia in Katowice. This work was supported by a non-commercial research grant from the Medical University of Silesia (WNM PTN-1-053/N/0/K). 

### 2.1. Echocardiography

TTE was performed according to the current American Society of Echocardiography (ASE) guidelines, using a commercially available two-dimensional imaging system (General Electric company Vivid e9, Milwaukee, WI, USA) [1]. Parameters were obtained and analyzed by 2 experienced sonographers blinded to patient data and CMR parameters. LVEF was calculated using Simpson’s two-dimensional biplane method (without ultrasound contrast), and left-ventricle mass (LVM) was estimated using the linear method and Cube formula. All the measurements, including LVM, left-ventricle hypertrophy (LVH), and left-ventricle remodeling type, were based on the recommendation of the ASE [1].

### 2.2. Cardiovascular Magnetic Resonance

The CMR images were acquired by the 1.5T system (GE Optima MR450w, Wauwatosa, GE Healthcare, Wauwatosa, WI, USA) with a dedicated phased-array cardiac coil, and they were analyzed using cardiac software (Cardiac VX 1.1.0, GE Healthcare, Chicago IL, USA). The CMR study protocols were used according to the guidelines [2]. The images used to evaluate the LV parameters and type of remodeling were obtained using a non-contrast part of the examination with multi-planar cine steady-state free precession (SSFP) acquisition. Cardiac chamber volumes and functions were analyzed with SSFP in several planes, including 2- and 4-chamber, orthogonal left ventricular outflow tract, and parallel short-axis planes covering both the atria and ventricles. The typical scan parameters used in the study were the time to echo/time of repetition (TE/TR) of 1.9/4.3 ms, slice thickness of 4–8 mm (no inter-slice gap), and temporal resolution of 30–40 ms. The SSFP planes for the atrioventricular prosthesis and ascending aorta were placed perpendicular to the aortic root. The left and right-ventricle (RV) parameters (volume and mass) were traced manually in each of the patients to provide the best precision. Papillary muscles were included in the left-ventricle chamber volume and excluded from LVM estimation.

### 2.3. Left-Ventricle Characteristics

**Figure 2:**
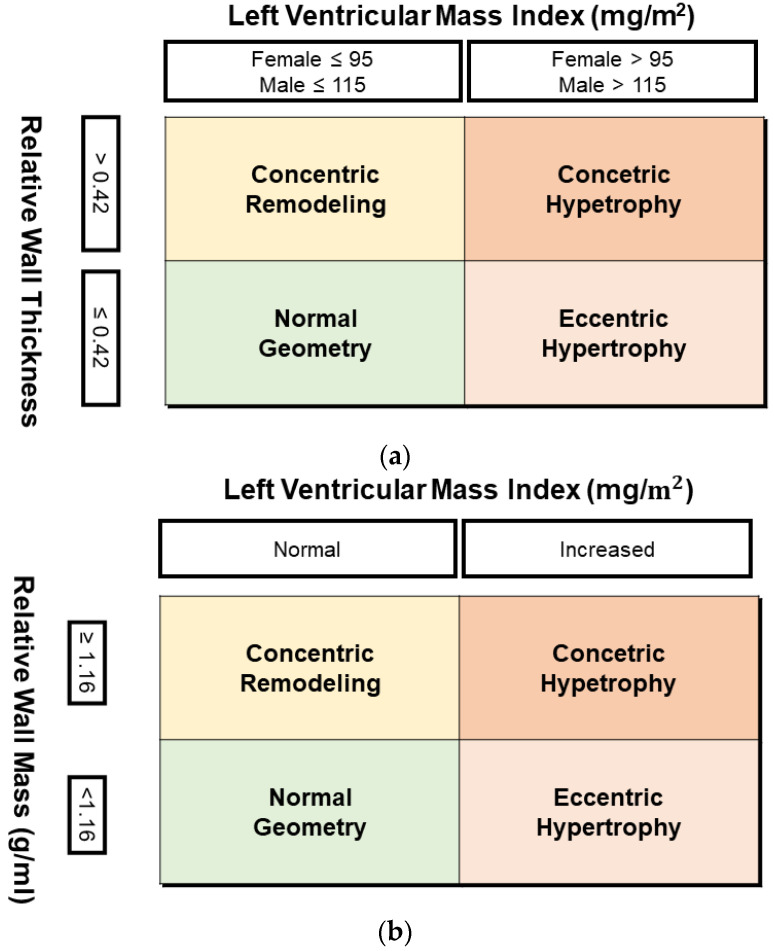
(**a**) Left-ventricle remodeling based on transthoracic echocardiography parameters. (**b**) Left-ventricle remodeling based on cardiac magnetic resonance.

LV parameters (mass and volume) were indexed to the body surface area (BSA) in both TTE and CMR. BSA was calculated using the Dubois and Dubois method [14]. Afterward, patients were categorized into predefined LV hypertrophy and remodeling patterns using reference values of LVM index (LVMi) and relative wall thickness (RWT) for TTE (Figure 2a) and LVMi and relative wall mass (RWM) for CMR (Figure 2b).

The normal reference range for LVMi assessed in TTE was 43–95 g/m^2^ for women and 49–115 g/m^2^ for men [1,3]. The RWT in TTE was calculated using the following formula, including the double diameter of posterior wall thickness divided by LV end-diastole diameter. The normal reference range for RWT in TTE was 0.22–0.42 for women and 0.24–0.42 for men, regardless of BSA [1,3].

The normal reference range for LVMi assessed in CMR was based on recent recommendations: 30–59 g/m^2^ for women and 36–75 g/m^2^ for men [15]. The RWM assessed in CMR was equivalent to the RWT evaluated in TTE and was calculated as the ratio of LVM and LV end-diastolic volume [16]. The upper normal range for RWM used in the study was 1.16, as presented in the previous study with healthy volunteers without coronary artery disease, hypertension, aortic stenosis, or other heart diseases [16].

The LV systolic function was classified based on the LVEF. The LVEF was calculated from LV end-diastolic volume (LVEDV) and LV end-systolic volume (LVESV) using the two-dimensional biplane method of disks (Simpson’s method) [3].

Given the baseline LVEF, the patient’s systolic function was classified as normal (preserved) ejection fraction (PEF) (LVEF ≥ 50%), mid-range ejection fraction (mrEF) (LVEF 40–49%), and reduced ejection fraction (REF) (LVEF < 40%) [17]. 

### 2.4. Statistical Analysis 

Variable normal distribution was analyzed with the Kolmogorov–Smirnov test. All TTE and CMR parameters were compared within the subgroups using *t*-tests for normally distributed continuous variables (Student’s *t*-test). The chi-squared test was used to compare the proportions between two subgroups. Associations between parameters were assessed using Pearson correlation analysis for parametric variables. A value of *p* < 0.05 was considered to be statistically significant. Statistical analysis was undertaken using Medcalc software (version 19.1, Osten, Belgium).

## 3. Results

### 3.1. Patient Population

The final study group included 202 patients (138 males) with a mean (standard deviation [SD]) age of 52.4 (16.3). Patient clinical characteristics are presented in Table 1. There were 28 subjects with CAD and preserved LVEF in Group 1 (14%), 64 patients with primary AR or PVL-AVR in Group 2 (32%), 80 individuals with HF in Group 3 (40%) and 30 patients with cardiomyopathies in Group 4 (15%).

### 3.2. Left-Ventricle Function and Remodeling

The following parameters of LV were assessed and compared between both modalities: LVMi, LVEDV, and LVEF. The mean LVMi values calculated in TTE were significantly larger (127.1 (44.5) g/m²) compared to the LVMi assessed in CMR (77.1 (26.2) g/m²; *p* < 0.001) in the study group. The TTE LVMi was also significantly larger in each of the subgroups compared to CMR.

The LVEDV assessed in echocardiography was underestimated in all the study patients (78.6 (43) mL vs. 100.5 (39) mL; *p* < 0.0001) and subgroups except for a statistical trend in patients with CM (*p* = 0.07) (Table 2).

There were no statistically significant differences in LVEF between TTE and CMR in the study group and the subgroups (Table 2). However, the great majority of the study patients had preserved LVEF.

Despite several differences in LV parameters, there were significant associations between both modalities in LVMi (r = 0.6; *p* < 0.0001), LVEDV (r = 0.65; *p* < 0.0001), or LVEF (r = 0.75; *p* < 0.001).

Moreover, patients with increased LVMi could have either concentric or eccentric hypertrophy based on RWT in echocardiography or RWM in CMR. Echocardiography showed a significantly higher rate of LVH compared to CMR (56% vs. 34%; *p* < 0.001).

The results of TTE RWT suggested that 90 patients (RWT ≤ 0.42) would have eccentric hypertrophy, and 26 patients would have concentric hypertrophy (RWT > 0.42). However, CMR assessment showed that 56 patients (RWM < 1.16) had eccentric hypertrophy, and only 1 patient (RWM ≥ 1.16) had concentric hypertrophy. Patients with normal LVMi and increased RWT or RWM had concentric remodeling of LV, which was found in 39 subjects when assessed by TTE and in 21 subjects when assessed in CMR (20.7% vs. 9.4%, *p* = 0.003).

**Figure 3:**
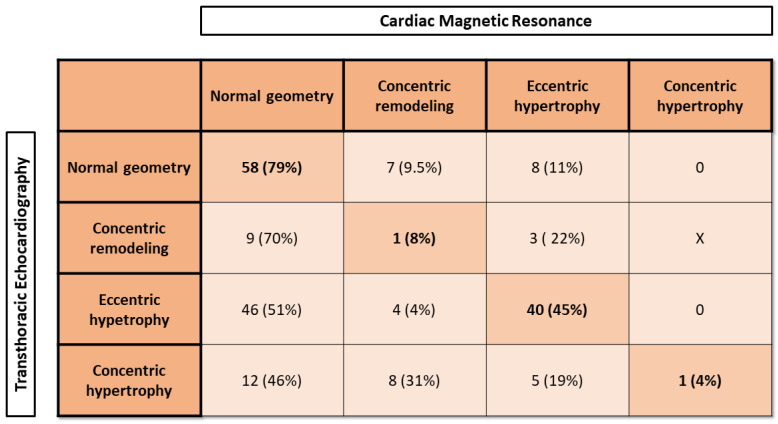
Left-ventricle remodeling type in transthoracic echocardiography and cardiac magnetic resonance.

The conclusions of LV remodeling in TTE and CMR are presented in Figure 3. While the best agreement was found in patients with normal LV geometry in TTE (79%), eccentric hypertrophy was confirmed in CMR in 45%. There was a very poor agreement between TTE and CMR in both phenotypes of LVH. Moreover, the differences in LV parameters are very important in Subgroups 2 (AR), 3 (HF), and 4 (CM). The CMR showed a significantly larger LVEDV in all patients with AR or PVL-AVR (Table 2). Moreover, CMR showed either a moderate dysfunction (2 patients with LVEF ≥ 50% in TTE) or preserved systolic function of LV (8 patients with LVEF < 50% in TTE).

In the subgroup with HF, both modalities showed either LVEF ≥ 35% (30 patients) or LVEF < 35% (40 patients). However, CMR showed a different degree of systolic dysfunction in 10 patients compared to TTE (LVEF < 35% in 6 patients and EF ≥ 35% in 4 patients). Moreover, CMR and TTE provided similar conclusions on the category of HF and systolic dysfunction–PEF, mrEF, or REF only in 55 patients (68%). CMR results changed the category of LV systolic function in 25 cases (32%).

The type of LV remodeling is especially important in patients within Group 4 (CM). Given the differences in LV parameters between both modalities, half of the cases (15 patients) showed divergent types of LV remodeling in TTE and CMR. 

## 4. Discussion

Our study compared two modalities in assessing the LV parameters, and it provided important results for clinical practice. There were four subgroups of patients representing the main indications for CMR.

First, we found that TTE systematically and significantly overestimated LVMi and underestimated the LVEDV compared to CMR. This resulted in discordant conclusions on the type of LV remodeling in half of the patients with a suspicion of cardiomyopathy. Finally, CMR changed the degree of LV systolic dysfunction in a considerable number of patients with heart failure, which would have an important impact on medical treatment. Our results provided novel findings on the incremental value of CMR in the assessment of LV remodeling.

Only a few studies have compared LV parameters obtained in TTE and CMR. They provided consistent results suggesting that TTE underestimated both LVEDV and LVESV. However, results on LVEF in both modalities were mostly discrepant [18,19,20,21]. The clinical consequences of those differences between TTE and CMR may result in a different approach to pharmacotherapy or device therapy (implantable cardioverter defibrillator (ICD) or cardiac resynchronization therapy (CRT)) in patients with HF [17]. Most of those studies included patients with preserved LVEF. However, other studies have suggested the superior role of CMR in the assessment of LVEF and the selection of patients for device implantation [22,23]. The precise quantification of LVEF is one of the major cardiac parameters, which is important for clinical decisions, including risk stratification, therapy, and clinical prognosis [8,9,10,17,18].

We found that CMR changed the conclusions on the degree of systolic dysfunction (below or above the indication for ICD) in 10 patients and the category of LV systolic dysfunction even in 25 cases.

There were systematic differences between both modalities in both LV mass and volume. While the LVEDV was mostly lower, the estimated mass was significantly overestimated in TTE compared to CMR. We found unacceptable significant differences in LVM between both modalities, which, in our opinion, undermines the practical value of the LVM formula in echocardiography. These differences in LV parameters would also have a significant effect on the conclusions related to the type of LV remodeling. We showed a modest or even poor agreement between the modalities in the phenotype of LV remodeling. The differences in the LV parameters between both modalities are mostly explained by the limitations of the echocardiography and suboptimal visibility of LV endocardial borders. The calculation of LV parameters in echocardiography is based on linear measurements and the assumption of left-ventricle geometry. Therefore, even a small difference in a linear measurement or a real LV shape results in a large difference in calculated LV mass in echocardiography [24]. CMR offers nearly unlimited visibility of real endocardial borders of both ventricle chambers and their geometry. Therefore, CMR provides a real LV mass instead of assumptions and mathematical formulas. Three-dimensional echocardiography is a novel option available in echocardiography, which shows a real shape of LV and even a three-dimensional (3D) reconstruction [25]. However, it has the same limitation as two-dimensional TTE as it needs an optimal visualization of cardiac chambers, which would not be available in a substantial number of patients [26]. Moreover, tachyarrhythmias and irregular heart rhythms significantly hinder the precise calculation of LV parameters in all imaging techniques, including computer tomography in modern models and updated software.

There is no simple point differentiating physiological adaptation and maladaptive LV remodeling. Mayala et al. published a systematic review of studies using CMR in a work-up of LV cardiomyopathies. It found that CMR has a high sensitivity, specificity, and positive predictive value in diagnosing different types of cardiomyopathies [27]. CMR was also found to be efficient in the differential diagnosis between ischemic and non-ischemic cardiomyopathies and in establishing the etiology of non-ischemic cardiomyopathy [28]. Moreover, CMR provides more precise results with a better value for patient prognosis compared to TTE [29]. CMR was demonstrated to improve accuracy in the assessment of LVM and LVH classification [30]. Precise diagnostic methods predicting outcomes are essential to improving and guiding therapy in patients with LV dysfunction.

Accurate quantification of AR severity and LV dimensions play a crucial role in the recommendation of aortic valve replacement (AVR) in asymptomatic patients with severe AR with a reduced LVEF or increased LV dimensions [18]. We found that TTE underestimated the degree of LV enlargement in patients with AR or AVR-PVL, which is an important factor for further treatment. Neisius et al. also showed a greater LV end-diastolic diameter (EDD) and end-systolic diameter in CMR compared to the TTE in patients with AR [31]. Puntmann et al. found a better correlation between CMR and TTE parameters of LV in a long axis rather than a short axis of chambers [32]. Recent guidelines have confirmed that LV enlargement and LVEF are crucial for clinical decisions and the time of intervention in patients with AR [9]. European Society of Cardiology guidelines for the management of valvular heart disease recommend particular threshold values of LV EDD and LVEF for interventions in severe AR, but those values are not dependent on the imaging modality [9]. Most studies that provided current cut-off values for interventions were based on echocardiography. However, our results showed important differences between TTE and CMR in a considerable number of patients. Those differences might have a direct impact on the conclusion, and it is an added value of multimodality cardiac imaging. 

Heart failure is the most important clinical indication for CMR. We showed that CMR changed the degree and the category of LV systolic dysfunction in 32% of cases with HF, and it also changed the indications for ICD implantation in the primary prevention of sudden cardiac death in 12% of our study patients. As expected, TTE and CMR showed the greatest agreement in patients with preserved LVEF and no significant remodeling. The current guidelines for the treatment of HF provide indications for selected pharmacotherapy, ICD, and CRT implantation based on the LVEF and clinical conditions [17,33]. Despite the superior quality of CMR in evaluating the LVEF, 2D TTE is still the most commonly used technique in routine practice for the evaluation of LV systolic function, and consecutive guidelines recommend TTE. However, CMR provides a better prognostic stratification and prediction of major adverse cardiac events, especially when CMR LVEF was combined with late gadolinium enhancement compared to TTE LVEF alone [34]. 

### 4.1. Clinical Implications

TTE is a principal imaging tool that is available and used in everyday clinical practice, and it is sufficient for therapeutic decisions in most cases. However, we showed that even a non-contrast CMR offers a superior diagnostic value, especially in diseased LV chambers. The greater the degree of LV remodeling and dysfunction, the greater the benefit from CMR. This suggests that CMR should be implemented into routine clinical practice for patients with cardiovascular conditions affecting LV volume, geometry, and shape. Moreover, patients with a limited quality of echocardiography or TTE parameters between the two categories of LVEF or heart-valve regurgitations (moderate to severe) should also be scheduled for CMR. However, CMR has some limitations in wide clinical use, mainly due to the limited availability of scanners and experienced teams, time of acquisition and analysis, or safety issues related to ferromagnetic metal devices. Nevertheless, CMR should have a strong place in routine clinical practice. 

### 4.2. Limitations

First, TTE and CMR were not performed on the same day. Although patients were clinically stable during the period between the two studies, some clinical changes may have occurred. Second, we did not include a 3D echocardiography, which may offer multidimensional images free of linear assumptions and could improve the evaluation of the LV cavity. However, 3D TTE is still largely dependent on acoustic window and cardiac chamber visualization and may not add clinical value. Therefore, a 2D TTE is still widely used in routine clinical practice. Third, a great majority of subjects had preserved LVEF, and there were no statistically significant differences in LVEF assessed in TTE or CMR in the study group or the subgroups. Those results may be limited by the sample size and clinical characteristics of patients. Finally, all the parameters were indexed to BSA, which seems to underestimate the prevalence of LVH in obese and some overweight hypertensive patients [35,36].

## 5. Conclusions

TTE significantly overestimates LVMi and underestimates LVEDV compared to CMR. This results in a significant disagreement between both modalities regarding the type of LV remodeling that impacts clinical decisions. CMR reclassified the degree of HF and the type of LV remodeling in patients with AR or AR-PVL. TTE and CMR are complementary but not strictly comparable methods for the assessment of LV mass, LV hypertrophy, and remodeling type. Further studies should assess the prognostic value of these differences between the modalities.

## Figures and Tables

**Table 1:** Study group clinical characteristics.

Characteristics	All Patients Mean (SD) or Number (%) *n* = 202
Age, years, mean (SD)	52.4 (16.3)
Males, *n* (%)	138 (68)
BMI, kg/m^2^, mean (SD)	27.8 ± 5.3
Obesity, *n* (%)	60 (30)
BSA, mean (SD)	1.94 (0.2)
Hypertension (%)	64 (31.5)
Diabetes mellitus (%)	29 (14)
Dyslipidemia (%)	96 (47)
Chronic kidney disease (%)	36 (18)

BMI—Body mass index, BSA—Body surface area, SD—standard deviation. Obesity defined as BMI ≥ 30 kg/m².

**Table 2:** Left-ventricle measurements were assessed using transthoracic echocardiography and cardiovascular magnetic resonance.

	TTE LVMI (g/m^2^), Mean (SD)	CMR LVMI (g/m^2^), Mean (SD)	*p* Value	TTE EDV (mL), Mean (SD)	CMR EDV (mL), Mean (SD)	*p* Value	TTE LVEF (%), Mean (SD)	CMR LVEF (%), Mean (SD)	*p* Value
All patients	127 (44.5)	77.1 (26.2)	*p* < 0.001	78.6 (43)	100.5 (39)	*p* < 0.0001	47 (14)	50.2 (16)	*p* = 0.1
Group 1 CAD	91 (21.1)	64.7 (14.2)	*p* = 0.04	65.4 (25.9)	86.1 (21)	*p* = 0.004	54.5 (9.9)	54.7 (14.5)	*p* = 0.95
Group 2 AR	132 (42)	78.6 (18.4)	*p* < 0.001	72.9 (46)	101.9 (41.2)	*p* = 0.0003	56.15 (8.5)	57.9 (11.7)	*p* = 0.33
Group 3 HF	130.1 (32)	79.2 (27.4)	*p* < 0.001	94.5 (46.1)	111.3 (43.4)	*p* = 0.045	37.1 (14)	39.9 (15.4)	*p* = 0.21
Group 4 CM	135 (71)	75.3 (40.2)	*p* = 0.003	69.1 (24.1)	82.7 (23.9)	*p* = 0.07	52.3 (11.6)	57.4 (13)	*p* = 0.11

CMR—Cardiac magnetic resonance, EDV—End-diastole volume, LVEF—Left-ventricle ejection fraction, LVMI—Left-ventricle mass index, SD—standard deviation, TTE—Transthoracic echocardiography, Group 1—chronic coronary artery disease with preserved left-ventricle ejection fraction and without significant valve disease (CAD); Group 2—native aortic valve regurgitation or paravalvular leak after aortic valve replacement (AR); Group 3—heart failure (HF); Group 4—cardiomyopathies (CM).

## Data Availability

The study data that support the findings of this study are available on request from the corresponding author.

## Abbreviations

AR	aortic regurgitation
ASE	American Society of Echocardiography
AVR	aortic valve replacement
BSA	body Surface area
CAD	coronary artery disease
CM	cardiomyopathy
CMR	cardiovascular magnetic resonance
CRT	cardiac resynchronization therapy
EDD	end-diastolic diameter
EDV	end-diastolic volume
EF	ejection fraction
ESV	end-systolic volume
HF	heart failure
ICD	implantable cardioverter defibrillator
LV	left ventricle
LVH	left-ventricle hypertrophy
LVM	left-ventricle mass
LVMi	left-ventricle mass index
mrEF	mid-range ejection fraction
PEF	preserved ejection fraction
PVL	paravalvular leak
REF	reduced ejection fraction
RV	right ventricle
RWM	relative wall mass
RWT	relative wall thickness
SSFP	steady-state free precession
TE	time of echo
TR	time of repetition
TTE	transthoracic echocardiography
